# Cortical Ribboning as a Key MRI Finding in Wernicke’s Encephalopathy With Altered Mental Status

**DOI:** 10.7759/cureus.79279

**Published:** 2025-02-19

**Authors:** Soutarou Taguchi, Takahiro Nakura, Manabu Doyu, Hidemoto Saiki

**Affiliations:** 1 Parkinson's Disease Advanced Therapy Center, Aichi Medical University Hospital, Nagakute, JPN; 2 Neurology, Aichi Medical University, Nagakute, JPN

**Keywords:** altered mental state, cortical ribboning, malnutrition, mri images, multiple system atrophy (msa), thiamine or vitamin b1 deficiency, wernicke’s encephalopathy (we)

## Abstract

Wernicke’s encephalopathy (WE) is associated with thiamine (vitamin B1) deficiency and may lead to mental status changes, ophthalmoplegia, and ataxia. While treatment is simple, delayed diagnosis can have serious consequences, making early detection essential. However, the complete triad of symptoms is rarely seen. When mental status changes occur, physical examination may be limited, making WE harder to identify without other characteristic signs. Furthermore, measuring blood thiamine levels is not always immediately possible, adding to the challenge of diagnosis. We encountered a case of WE presenting with mental status changes, where cortical ribboning appeared as a significant MRI finding. A 77-year-old male had experienced slowness of movement for approximately one year and was diagnosed with multiple system atrophy (MSA). He had difficulty eating; thus, he preferred soft rice porridge. He developed acute mental status changes, and a brain MRI revealed prominent cortical ribboning in both frontal cortices along with subtle medial thalami. The patient received intravenous thiamine, leading to dramatic recovery of mental status, and subsequent MRI follow-up showed near-complete resolution of both cortical and thalamic lesions. Cortical ribboning, which played a key role in this patient, is recognized as an atypical MRI finding of thiamine deficiency and is suggested to be associated with altered mental status in WE. However, to the best of our knowledge, no literature clearly lists thiamine deficiency as a differential diagnosis for cortical ribboning in patients with mental status changes. Recognizing this key imaging finding is crucial in differentiating WE in patients with mental status changes. Preventing the potentially fatal outcome of WE is of paramount importance. Patients with dysphagia tend to prefer soft foods, which may result in reduced thiamine intake. Our experience reaffirms the importance of a well-balanced diet in MSA patients with dysphagia.

## Introduction

Wernicke’s encephalopathy (WE) is a disease caused by thiamine (vitamin B1) deficiency and is classically characterized by a triad of mental status changes, ophthalmoplegia, and ataxia. While its treatment is simple, delayed diagnosis can result in a fatal outcome, highlighting the need for prompt identification [1]. However, it is important to note that only 16% of patients exhibit the classic triad [2], which makes diagnosis challenging in many cases. Particularly, when altered mental status is present, limitations in physical examination make it difficult to suspect WE based on the lack of other symptoms of the typical triad. Moreover, blood thiamine levels are often not immediately available, further complicating the diagnosis of WE. Therefore, using brain imaging for patients with mental status changes is valuable in diagnosing WE and ruling out differential diagnoses. In WE, brain MRI typically reveals symmetrical high-signal lesions in the thalamus and periaqueductal region on diffusion-weighted imaging (DWI), T2-weighted imaging, and fluid-attenuated inversion recovery (FLAIR) imaging. However, these typical findings are observed in only about 60% of cases [3]. An atypical MRI finding in WE, cortical ribboning (a ribbon-like signal change in the cerebral cortex), has been reported in approximately 20% of cases with altered mental status [3-5]. However, to the best of our knowledge, no literature clearly lists thiamine deficiency as a differential diagnosis for cortical ribboning in patients with mental status changes [6]. Here, we report a case of WE with mental status changes, in which cortical ribboning was a key MRI finding.

## Case presentation

**Figure 1:**
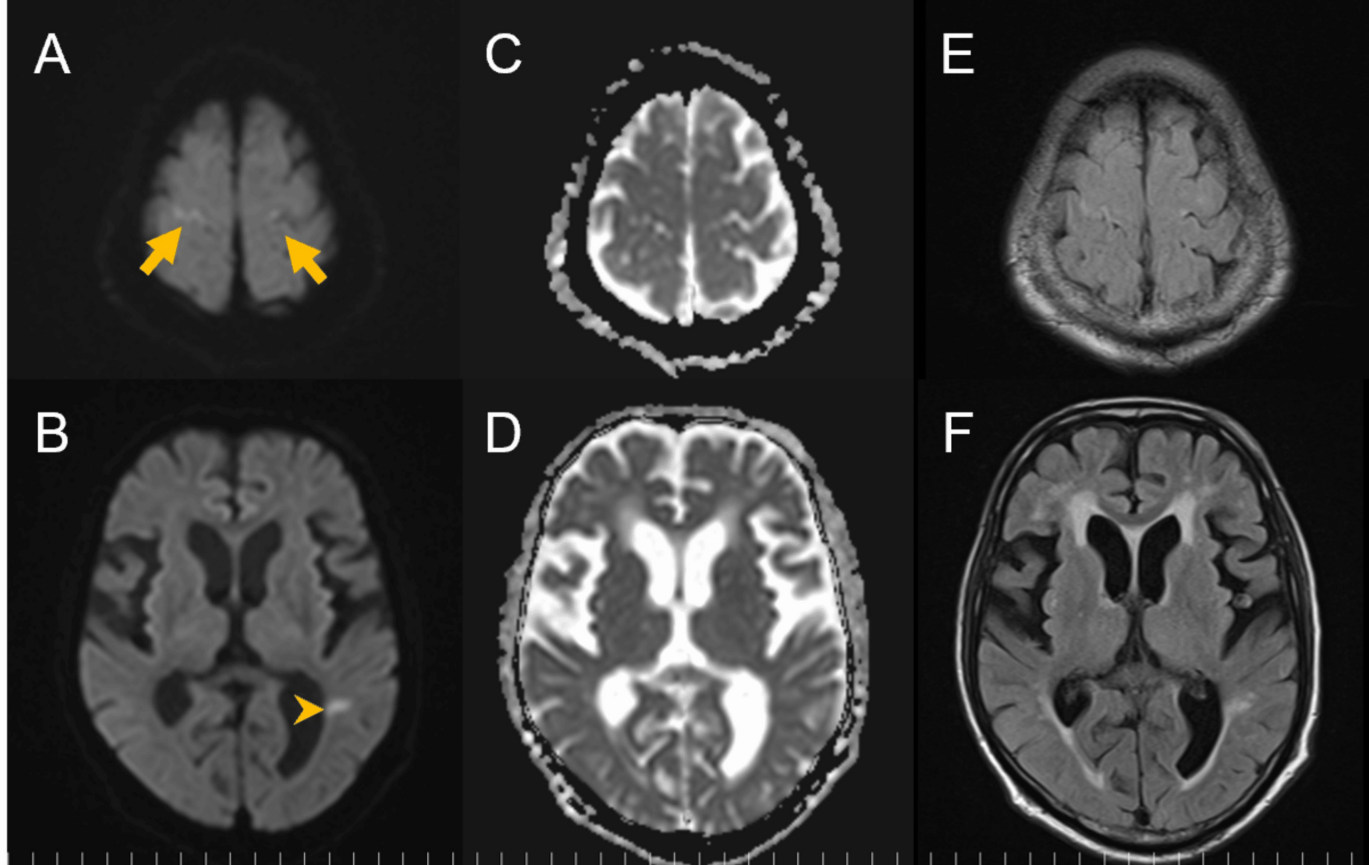
Brain MRI three days before the onset of altered mental status (A, C, E) The frontal cortex (arrow) that was later recognized as abnormal shows slightly high signal intensity on DWI, decreased ADC, and slightly high signal on FLAIR, but was overlooked at this stage. (B, D, F) A small lesion (arrowhead) in the left parietal subcortical area shows high signal intensity on DWI, reduced ADC, and high signal on FLAIR. This lesion was suspected to be ischemic but was considered unrelated to the patient's hypobulia. ADC, apparent diffusion coefficient; DWI, diffusion-weighted imaging; FLAIR, fluid-attenuated inversion recovery

A 77-year-old right-handed Japanese male was prescribed duloxetine and lemborexant by a psychiatrist for depression. He had no significant family history. For approximately one year, he had been experiencing slowness of movement and difficulty eating; thus, he preferred soft rice porridge. He had no history of excessive alcohol consumption. To investigate the cause of his slowness, he was admitted to our center and was diagnosed with multiple system atrophy (MSA) based on the criteria of the Movement Disorder Society. A few days before discharge, he exhibited mild hypobulia, but the physical examination revealed rigidity, bradykinesia, postural instability, and myoclonus in both hands, all of which were consistent with MSA symptoms. There was no worsening of ataxia or new neurological abnormalities, including ophthalmoplegia. Whole-body CT, blood tests, and rapid flu/COVID-19 tests were unremarkable. Brain MRI revealed a small lesion in the left parietal subcortical area, showing high signal intensity on DWI and FLAIR with decreased apparent diffusion coefficient (ADC). This lesion was suspected to be ischemic but was considered unrelated to his hypobulia. The subtle abnormalities in the frontal cortex, later recognized as significant, were overlooked at this stage (Figure 1).

After a nine-day hospitalization, he was discharged home walking. Two days after discharge, he developed acute mental status changes and was transported to the emergency department.

**Figure 2:**
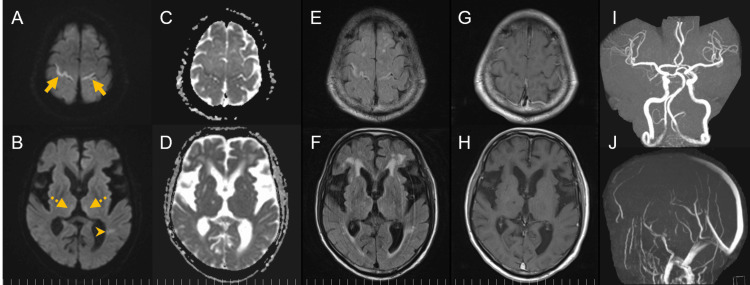
Brain MRI during altered mental status (A, C, E, G) Prominent cortical ribboning lesions (arrows) are observed in both frontal cortices, showing high signal intensity on DWI, reduced ADC, and high signal on FLAIR, with no gadolinium enhancement. (B, D, F, H) Less prominent lesions (dashed arrows) are observed in the medial thalami bilaterally, showing a similar pattern. The previously noted left parietal subcortical lesion (arrowhead), which had been identified a few days earlier, shows an unchanged high signal on DWI and FLAIR, but the reduction in ADC has resolved, suggesting that it represents a time-dependent change in cerebral ischemia. (I, J) Magnetic resonance angiography/venography showed no arterial or venous occlusions. ADC, apparent diffusion coefficient; DWI, diffusion-weighted imaging; FLAIR, fluid-attenuated inversion recovery

His Glasgow Coma Scale (GCS) score was E3V1M5, blood pressure was 176/105 mmHg, heart rate was 81 bpm (regular), body temperature was 37.4°C, and SpO2 was 98% (room air). His pupils were 3.0 mm bilaterally; the right pupillary light reflex was difficult to evaluate due to cataracts, but the left pupil constricted briskly. His eye position was orthophoric, and extraocular movements were full. In addition to the previously noted in both hands, myoclonus was also observed in his mouth. There were no convulsions. Given the possibility of nonconvulsive status epilepticus, intravenous diazepam (5 mg) was administered, slightly reducing myoclonus but not improving mental status. Brain MRI revealed prominent cortical ribboning in both frontal cortices, with high signal intensity on DWI and FLAIR, reduced ADC, and no gadolinium enhancement. Less prominent but similar signal changes were observed in the medial thalami bilaterally. The previously noted left parietal subcortical lesion remained unchanged on DWI and FLAIR but no longer showed ADC reduction, suggesting that it represents a time-dependent change in cerebral ischemia. There were no abnormalities in the periaqueductal region, and MR angiography/venography showed no arterial or venous occlusions (Figure 2).

Whole-body CT and rapid flu/COVID-19 tests were unremarkable. Blood tests showed no hypo/hyperglycemia, liver dysfunction, renal dysfunction, or electrolyte imbalances. CSF analysis revealed a slight increase in protein but normal cell counts and glucose levels. Spinal fluid PCR test did not detect any pathogens associated with encephalopathy, including herpes simplex virus, varicella-zoster virus, cryptococcus, or *Mycobacterium tuberculosis *(Table 1).

**Table 1:** Laboratory results and reference ranges

Parameter	Patient value	Reference range	Unit
Blood			
White blood cells	8,000	3,300-8,600	/uL
Hemoglobin	12.3	13.7-16.8	g/dl
Platelet count	318	158-348	10^3^/ul
Urea nitrogen	30.2	8.0-20.0	mg/dl
Creatinine level	1.01	0.65-1.07	mg/dl
Sodium level	142	138-145	mmol/l
Ionized calcium level	1.11	1.12-1.33	mmol/l
Total bilirubin	1.31	0.40-1.50	mg/dl
Aspartate aminotransferase	22	13-30	U/l
Alanine aminotransferase	11	10-42	U/l
Glucose	100	73-109	mg/dl
CRP	2.53	<0.14	mg/dl
Thyroid-stimulating hormone	0.356	0.610-4.230	uIU/ml
Free T4	1.35	0.83-1.53	ng/dl
Fibrinogen/fibrin degradation products	3.1	<5.00	ug/ml
Vitamin B1	16	24-66	ng/ml
Carbon dioxide	44.3	43-53	mmHg
Cerebrospinal fluid			
Cell	0	<5	/ul
Protein	47	10-40	mg/dl
Glucose	57	50-80	mg/dl

**Figure 3:**
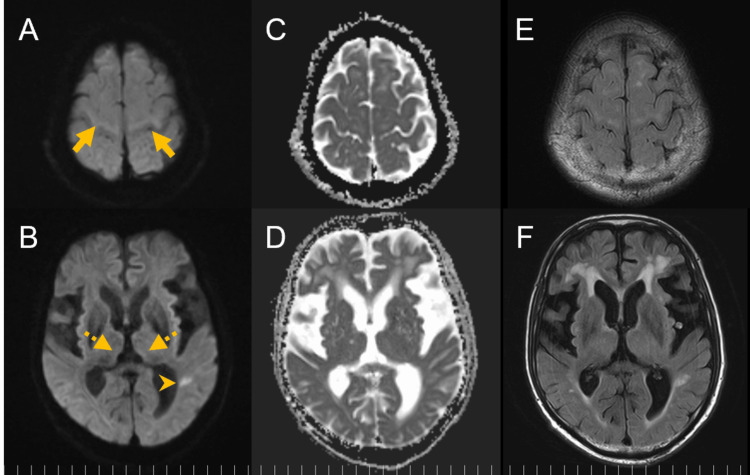
Brain MRI 32 days after the onset of altered mental status, by which time the patient’s mental status had recovered (A, C, E) The cortical ribboning in both frontal cortices (arrows) has almost completely resolved. DWI and ADC are normalized, and only a slight high signal is observed on FLAIR. (B, D, F) Abnormalities in the medial thalami bilaterally (dashed arrows) are no longer prominent. The left parietal subcortical lesion (arrowhead) still shows a high signal on DWI and FLAIR, with normal ADC, suggesting that it represents a time-dependent change in cerebral ischemia. ADC, apparent diffusion coefficient; DWI, diffusion-weighted imaging; FLAIR, fluid-attenuated inversion recovery

The patient received intravenous thiamine (1,500 mg/day), leading to dramatic recovery of mental status within hours. Two weeks after emergency admission, blood tests conducted prior to thiamine administration confirmed thiamine deficiency (16 ng/ml) and ruled out thyroid dysfunction, anti-myelin oligodendrocyte glycoprotein-associated antibody positivity, as well as pathogenic organisms in both CSF and blood cultures. Cortical ribboning and medial thalamic lesions gradually resolved, and an MRI performed 32 days after the onset of altered mental status showed near-complete resolution. However, the left parietal subcortical lesion persisted (Figure 3).

The patient exhibited only MSA-related symptoms thereafter and was discharged 33 days after admission.

## Discussion

In this patient, the diagnosis of WE was made based on the history, symptoms, and clinical improvement due to thiamin supplementation [1]. There were no historical, symptomatic, or laboratory findings suggestive of other causes of altered mental status, such as alcohol intoxication, hypo/hyperglycemia, uremia, renal dysfunction, electrolyte imbalance, meningoencephalitis, hypoxia, hypercapnia, hypo/hyperthermia, stroke, or epileptic seizures [7].

The MRI abnormalities in this patient played a key role in confirming the diagnosis of WE. The imaging findings demonstrated selective involvement of the cerebral cortical gray matter while sparing the adjacent white matter, a pattern known as cortical ribboning. When ribboning lesions exhibit hyperintensity on DWI and reduced ADC, they are considered to reflect cytotoxic edema of neurons located in the cortical gray matter. Reported causes of this phenomenon in the literature include cerebral infarction (including venous sinus thrombosis), Creutzfeldt-Jakob disease, herpes simplex virus infection, epileptic seizures, hypoglycemia, and mitochondrial diseases [6]. On the other hand, while cortical ribboning has been previously reported as an MRI finding in thiamine deficiency [4], to the best of our knowledge, no literature clearly lists thiamine deficiency as a differential diagnosis for cortical ribboning in patients with mental status changes [6]. As seen in our patient, WE often begin with mild and vague symptoms such as hypobulia, dizziness, and weakness. Even as the disease progresses, only 16% of patients exhibit the classic triad [2]. Particularly in cases of altered mental status, limitations in physical examination make it difficult to suspect WE based on incomplete symptoms. Moreover, blood thiamine levels are often not immediately available, further complicating the diagnosis of WE. Therefore, using brain imaging in patients with mental status changes is valuable in diagnosing WE and ruling out differential diagnoses. The typical MRI findings of WE consist of symmetrical lesions around the thalamus and the periaqueductal region, but these findings are observed in only about 60% of cases [3]. Since cortical ribboning has been suggested to be associated with altered mental status or a worse prognosis in WE [3-5], recognizing this key imaging finding is crucial in differentiating WE in patients with mental status changes where diagnosis is challenging.

Why thiamine deficiency causes cortical ribboning and how cortical ribboning in WE relates to the mechanisms underlying altered mental status remain largely unknown. To date, only a few studies have explored this issue, and their findings can be summarized as follows: (1) Thiamine deficiency leads to a reduction in glucose metabolism efficiency, resulting in lactic acidosis and subsequent cytotoxic edema [8]. (2) Regions with a high density of neurons and astrocytes, as well as active metabolic activity, are more vulnerable to damage. In WE, the typical lesions - such as those in the thalamus and periaqueductal region - are more susceptible to thiamine deficiency than the cortex [8]. (3) In WE patients with altered mental status, thalamic lesions are invariably present, whereas cortical lesions are observed in only approximately 20% of cases [4]. (4) The histopathological features of cortical ribboning and those of typical WE lesions in the thalamus and periaqueductal region are both characterized by necrosis and astrocytosis, with no fundamental differences between them [9]. (5) When cortical involvement is present, altered mental status is more severe, and the prognosis is worse compared to WE with thalamic lesions only [4]. Building on our observations and previous findings, investigating the incidence of ribboning in WE, its changes following thiamine supplementation, and the differences in the nature and severity of altered mental status depending on the presence or absence of cortical involvement may help clarify the relationship between thiamine deficiency-induced altered mental status and cortical damage. Such research could contribute to enhancing the diagnosis of WE, particularly in patients with altered mental status who have a poor prognosis. We hope that future research in this field will further advance our understanding. In particular, we anticipate that this important imaging finding, along with a well-defined underlying mechanism, will be recognized and incorporated into reviews and textbooks on altered mental status.

The left parietal subcortical lesion in this patient is considered to be an asymptomatic cerebral infarction that occurred independently of thiamine deficiency. This is based on the fact that its signal pattern differs from that observed in the frontal cortex and thalamus, which are considered to be attributable to WE; lesions adjacent to the lateral ventricle in this region have not been reported in WE [2]; and the time course of signal changes does not contradict that of cerebral ischemia.

Preventing the potentially fatal outcome of WE is of paramount importance. Patients with dysphagia, such as our case, tend to prefer soft foods that are easy to swallow, which may result in reduced thiamine intake. Rice, a staple food in many Asian diets, is often refined from brown rice, significantly reducing its thiamine content through the cooking process [1]. A study evaluating vitamin levels in MSA patients did not observe thiamine deficiency [10]; however, our experience reaffirms the importance of a well-balanced diet in MSA patients with dysphagia.

## Conclusions

We report a case of WE with mental status changes, in which cortical ribboning was a key MRI finding. Our findings suggest that cortical ribboning on MRI, especially in a patient with mental status changes, should prompt consideration of WE as a differential diagnosis.

Patients with dysphagia are at risk of developing dietary imbalances, which may lead to WE. Our experience highlights the importance of maintaining a well-balanced diet in patients with MSA who have dysphagia.

## References

[1] Sechi G, Serra A (2007). Wernicke's encephalopathy: new clinical settings and recent advances in diagnosis and management. Lancet Neurol.

[2] Harper CG, Giles M, Finlay-Jones R (1986). Clinical signs in the Wernicke-Korsakoff complex: a retrospective analysis of 131 cases diagnosed at necropsy. J Neurol Neurosurg Psychiatry.

[3] Zuccoli G, Pipitone N (2009). Neuroimaging findings in acute Wernicke's encephalopathy: review of the literature. AJR Am J Roentgenol.

[4] Fei GQ, Zhong C, Jin L (2008). Clinical characteristics and MR imaging features of nonalcoholic Wernicke encephalopathy. AJNR Am J Neuroradiol.

[5] Ota Y, Capizzano AA, Moritani T, Naganawa S, Kurokawa R, Srinivasan A (2020). Comprehensive review of Wernicke encephalopathy: pathophysiology, clinical symptoms and imaging findings. Jpn J Radiol.

[6] Pai V, Sitoh YY, Purohit B (2020). Gyriform restricted diffusion in adults: looking beyond thrombo-occlusions. Insights Imaging.

[7] Edlow JA, Rabinstein A, Traub SJ (2014). Diagnosis of reversible causes of coma. Lancet.

[8] Manzo G, De Gennaro A, Cozzolino A, Serino A, Fenza G, Manto A (2014). MR imaging findings in alcoholic and nonalcoholic acute Wernicke's encephalopathy: a review. Biomed Res Int.

[9] Yamashita M, Yamamoto T (1995). Wernicke encephalopathy with symmetric pericentral involvement: MR findings. J Comput Assist Tomogr.

[10] Chen D, Wan L, Chen Z (2022). Serum vitamin levels in multiple system atrophy: a case-control study. Front Aging Neurosci.

